# Effect of Honey, Coenzyme Q10, and β-Carotene/α-Tocopherol as Novel Additives in Rabbit-Sperm Cryopreservation Extender

**DOI:** 10.3390/ani13142392

**Published:** 2023-07-24

**Authors:** Jaume Gardela, Mateo Ruiz-Conca, Anna Palomares, Sergi Olvera-Maneu, Laura García-Calvo, Manel López-Béjar, Felipe Martínez-Pastor, Manuel Álvarez-Rodríguez

**Affiliations:** 1 Department of Animal Health and Anatomy, Faculty of Veterinary Medicine, Universitat Autònoma de Barcelona, 08193 Bellaterra, Spain; 2 College of Veterinary Medicine, Western University of Health Sciences, Pomona, CA 91766, USA; 3 Institute of Animal Health and Cattle Development (INDEGSAL) and Department of Molecular Biology (Cell Biology), Universidad de León, 24009 León, Spain; 4 Department of Animal Reproduction, National Institute for Agriculture and Food Research and Technology, Spanish National Research Council (INIA-CSIC), 28040 Madrid, Spain

**Keywords:** antioxidants, semen, sperm motility, proAKAP4

## Abstract

**Simple Summary:**

Rabbit sperm cryopreservation efficiency is still suboptimal. Post-thawing qualities, such as motility and membrane integrity, are severely impaired after the cryopreservation procedure. One strategy to increase cryopreservation efficiency is the use of novel extender components with cryoprotective effects. In the current study, we aim to test the use of honey, coenzyme Q10, and β-carotene/α-tocopherol as novel additives for rabbit-sperm cryopreservation. Additionally, we used, for the first time, proAKAP4 as a molecular marker candidate for sperm quality in rabbits. Our results demonstrated that adding 2.5% honey to the semen extender improved the post-thawing rabbit-sperm motility compared to higher concentrations of honey. In contrast, coenzyme Q10 was harmful to sperm motility after cryopreservation. Furthermore, the β-carotene/α-tocopherol neither improved nor worsened the post-thawing sperm motility compared to the base extender media. In conclusion, the cryopreservation protocols used in this study failed to preserve the fresh-sperm parameters, paving the way for additional studies to improve rabbit sperm cryopreservation protocols.

**Abstract:**

The effectiveness of rabbit-sperm cryopreservation is still below average compared to other domestic species. After the sperm cryopreservation process, post-thawing parameters like motility and membrane integrity are significantly compromised. The use of new extender constituents is an approach that can be used to improve the effectiveness of cryopreservation. Accordingly, we used honey (1.25, 2.5, 5, and 10%), coenzyme Q10 (100 and 200 μM), and β-carotene/α-tocopherol (500 μM/620 μM and 250 μM/310 μM) as candidate components for rabbit-sperm extenders during cryopreservation. Ejaculates from commercial adult rabbit bucks (*n* = 5) were cryopreserved using conventional freezing. Several post-thawing sperm parameters were assessed, including total motility, membrane integrity, viability, nuclear membrane integrity, acrosome reaction, and mitochondrial membrane potential and activation. Additionally, we performed hormonal analyses of the seminal plasma. Moreover, we analyzed the post-thawing levels of a molecular marker of sperm quality, proAKAP4, which was used in rabbits for the first time. Our findings showed that the 2.5% honey supplementation increased the post-thawing sperm motility (13.75 ± 3.75%) compared to the greater concentrations employed. However, the post-thawing motility was negatively affected by the coenzyme Q10 (0%, in both groups) but was not affected by the β-carotene/α-tocopherol supplementation (22 ± 18.15%, and 11.67 ± 10.17%). In conclusion, the cryopreservation protocols of this study did not help to maintain the sperm parameters after thawing. Further studies are required to identify novel protocols to mitigate the damage caused to rabbit sperm during cryopreservation.

## 1. Introduction

The relevance of artificial insemination (AI) in rabbits (*Oryctolagus cuniculus*) has increased over the years [1]. In rabbits, AI with fresh or refrigerated semen yields good fertility and prolificacy results but its use is conditioned by limited storage time [2]. Cryopreservation by freezing or vitrification procedures may help to keep the sperm cells indefinitely for use in assisted reproductive technologies. However, it is still a challenge to achieve the same success rates as with refrigerated semen [3]. For this reason, many studies have focused on improving sperm survival. The most widely used technique in these studies is sperm freezing as vitrification is still little developed in this species [4].

The cryopreservation process involves several steps, including cooling, freezing, and thawing, all inducing cellular membrane damage and reducing motility [5]. Throughout the cryopreservation, sperm cells are exposed to various stressors, such as drastic temperature changes, the formation of intracellular ice crystals, and the osmotic and toxic effects of the cryoprotective agents (CPA) used during the procedure [6,7]. The seriousness of these stresses varies among species; the stress derived from CPAs is one of the most important in rabbits since the composition of the sperm plasma membrane makes it resilient to temperature changes [8]. Current protocols result in 40–50% loss of functional sperm after the cryopreservation process [9], with high variability among individuals [10].

One of the strategies to cope, at least partly, with the detrimental effects of the cryopreservation process is the addition of antioxidant components to the extender media, which has been shown to be helpful in other species [11,12]. Therefore, this study focused on testing several protective agents for improving the cryopreservation of rabbit semen in standard extenders. Honey has drawn attention for this purpose. It is mostly a mixture of sugars and water, containing a rich blend of proteins and secondary metabolites [13], and is renowned for its antibacterial properties [14,15]. It has been previously used as a semen extender additive in rams [16,17], humans [18], stallions [19], bulls [20,21], and boars [22] but still untested in rabbits. Another candidate tested was a combination of β-carotene and α-tocopherol. β-carotenes are antioxidants involved in vitamin E recycling in the cell membranes, being one analog, Trolox, analyzed in sperm cryopreservation of different species [23,24,25,26]. Some carotenoids have been tested in several species, such as crocin, showing a strong antioxidant activity [27,28,29,30]. In addition, the coenzyme Q10 (CoQ10) is an essential component for electron transport in oxidative phosphorylation of mitochondria [31]. The CoQ10 is present at high levels in the spermatozoon as its reduced form, ubiquinol [32,33]. It has been successfully used for freezing stallion semen with contrasting results [34,35,36,37], whereas other authors showed that adding it to the horse-sperm freezing medium did not increase sperm motility [38]. In humans, CoQ10 has been used in the semen of oligospermic patients successfully reducing the number of reactive oxygen species and decreasing sperm damage during cryopreservation [39].

On the other hand, hormonal measurements in seminal plasma (SP) could help in the establishment of novel biomarkers [40], perhaps useful as cryopreservation efficiency predictors. In this sense, hormonal levels are also potential indicators of animal stress, controlled mainly by the adrenal cortex for the sympathetic–medullary–adrenal axis [41]. Cortisol is often associated with stress and it is functionally involved in controlling animals’ behavior together with metabolic, endocrine, and immune functions to ensure adequate coping strategies and wellbeing [42]. In addition, testosterone concentration is essential to maintain the auxiliary gland function of male animals, such as protein and fructose synthesis. Fructose in the SP is secreted by seminal vesicles, which are very sensitive to androgen stimulation [43]. Moreover, testosterone significantly decreased in rabbit bucks under heat-stress conditions [44] and incubation of small ruminants’ sperm before cryopreservation impairs sperm survival [45]. Lastly, the anti-Müllerian hormone (AMH), which plays an essential role in the maturation and differentiation of spermatogenic cells, is expressed in Sertoli cells and the AMH of the SP can provide direct information about spermatogenesis [46]. Moreover, the AMH could predict motile sperm recovery after semen cryopreservation in men with asthenozoospermic [47].

Finally, since changes in cryopreservation protocols are often subtle and do not reflect classical sperm-quality parameters, we used a novel test for assessing the functionality of the processed sperm samples. The proAKAP4 protein is a highly conserved molecule in spermatozoa [48,49,50] which is a sperm-specific member of the A-kinase anchor family of proteins [51]. This protein is only expressed in the postmeiotic phase of spermatogenesis at the round spermatid stage [49] and it has been proposed as a motility, morphology, fertility, and prolificacy biomarker [52], and even as a marker of freezability (equine [53,54]; bovine [55]; and ovine [56]), but there are no studies in rabbit yet.

Therefore, the main objectives of this study were to evaluate the protective effect of different extender supplements (honey, CoQ10, and β-carotene/α-tocopherol) for conventional freezing of rabbit sperm. To achieve that, we used different techniques, including the analysis of proAKAP4, a potential fertility marker with the ability to withstand the cryopreservation process in this species. We hypothesized that by using the supplements, the frozen/thawed rabbit sperm will keep success rates as seen with refrigerated semen.

## 2. Materials and Methods

### 2.1. Reagents

The chemicals used in the experiments were of analytical grade. Unless otherwise stated, all reagents were acquired from Sigma-Aldrich (St. Louis, MO, USA). GALAP was purchased from IMV technologies (L’Aigle, France) and Gent^®^ from Minitube Ibérica (Tarragona, Spain). The water-soluble β-carotene (10 mg/100 mg)–α-tocopherol (1 mg/100 mg) mixture was kindly donated by DSM (León, Spain). The raw wildflower bee honey was kindly donated by Laura García, obtained from hives of local bees (ES24002000007; Algadefe, León, Spain). No compositional analysis of this additive was performed. For all the experiments included in this study, a single batch of honey was used.

### 2.2. Animals and Housing Conditions

Five New Zealand White (NZW) adult rabbit bucks (from 6 to 7 months old), from the nucleus colony at the farm of the Institut de Recerca i Tecnologia Agroalimentaries (IRTA-Torre Marimon, Caldes de Montbui, Barcelona, Spain) and transported to the Servei de Granges at the Faculty of Veterinary Medicine, Universitat Autònoma de Barcelona, were included in this study. Each animal was individually housed in a cage (85 × 40 × 30 cm) equipped with plastic footrests, a feeder (restricted to 180 g/day of an all-mash pellet), and *ad libitum* access to water. Animals were kept under a controlled photoperiod of 16 h of light and 8 h of darkness, and a range of temperature between 20 and 26 °C, with a relative humidity of 60% to 75% maintained by a forced ventilation system. Animal husbandry and experimental handling were performed according to the European Directive 2010/63/EU, 22/09/2010 for animal experiments.

### 2.3. Experimental Design

Two independent experiments were performed in this study using the same adult rabbit bucks, collecting a single ejaculate from each male (*n* = 5).

In Experiment 1, we tested the effects of four concentrations of bee honey, as an egg yolk replacement, (1.25, 2.5, 5, and 10%) in a commercial fresh rabbit-semen storage medium (GALAP, IMV technologies, L’Aigle, France) supplemented with 5% dimethyl sulfoxide (DMSO) as a rabbit-sperm cryopreservation media. Additionally, we tested an egg yolk-based commercial semen extender containing glycerol (Gent^®^; Minitube Ibérica, Tarragona, Spain) and a standard sperm extender, TCG (Tris, citric acid, glucose, 20% egg yolk, and 5% DMSO), as candidates for rabbit sperm cryopreservation (Table 1). For Experiment 1, only total motility and membrane integrity were recorded.

In Experiment 2, we tested the effects of two concentrations of CoQ10 (100 and 200 μM) and two concentrations of β-carotene/α-tocopherol (500 μM/620 μM and 250 μM/310 μM) in a commercial fresh rabbit-semen storage medium (GALAP; IMV technologies, L’Aigle, France) supplemented with 20% egg yolk and 5% DMSO as a rabbit-sperm cryopreservation media (Table 1). Previous studies used concentrations of α-tocopherol at 2 mM and β-carotene 0.5 mM in the cryopreservation of rainbow-trout semen [57] and α-tocopherol at 4.8 mM for the cryopreservation of bull semen [58]. GALAP commercial fresh rabbit-semen storage medium supplemented with 20% egg yolk and 5% DMSO was used as a control.

All the reagents prepared for Experiments 1 and 2 are depicted in Table 1. The egg yolk was obtained from fresh chicken eggs. The recovered yolk was added to the required extenders and then clarified by centrifugation of 10,000× *g* for 20 min. After that, for the extenders that contain DMSO, a phase B was prepared at 10% DMSO, although the final concentration obtained was 5% DMSO after dilution (1:1) between phases A (extender base media) and B. The osmolality of the extenders used was measured using a freezing point osmometer (Osmomat 3000; Gonotec, Berlin, Germany). The measured osmolalities are within the range of similar commercial extenders used for cryopreservation procedures in different species [28,59].

### 2.4. Semen Samples Collection

All males started to be trained with an artificial vagina at 4.5 months of age. A commercial polyvinyl chloride artificial vagina (HUMECO, Huesca, Spain) containing water at 50 °C was used [60]. Samples were macroscopically assessed to discard insufficient quality criteria, including alterations in color and thickness. Ejaculates that contained urine and calcium carbonate deposits on visual inspection were discarded. The samples were transported to the laboratory within 10 min and kept at 38.5–39 °C, being evaluated under a microscope to evaluate the sperm motility and to discard individuals with low motility or subjective concentration.

Accepted samples were split into aliquots and immediately centrifuged for removing SP (500× *g*, 5 min, room temperature). The SP was checked for the absence of cells after the centrifugation and stored separately for hormonal analysis at −80 °C. The sperm pellets were extended using the reagents prepared for Experiment 1 or Experiment 2 (Table 1).

### 2.5. Hormonal Seminal Plasma Analyses

Cortisol, testosterone, and anti-Müllerian hormone (AMH) in SP were assessed by commercial enzyme immunoassay (EIA) kits. The Cortisol EIA kit (Cortisol ELISA KIT; Neogen^®^ Corporation, Ayr, UK) presented cross-reactivity with prednisolone (47.4%), cortisone (15.7%), 11-deoxycortisol (15.0%), prednisone (7.83%), corticosterone (4.81%), 6β-hydroxycortisol (1.37%), 17-hydroxyprogesterone (1.36%), deoxycorticosterone (0.94%), progesterone (0.06%), and all other steroids (<0.06%). The Testosterone EIA kit (Testosterone ELISA KIT; Neogen^®^ Corporation, Ayr, UK) presented cross-reactivity with testosterone glucuronide (16.12%), androstenedione (0.86%), bolandiol (0.86%), testosterone enanthate (0.13%), estriol (0.10%), testosterone benzoate (0.10%), estradiol (0.05%), dehydroepiandrosterone (0.04%), and all other steroids (<0.09%). No significant cross-reactivity or interference between AMH and analogs was observed in the AMH EIA kit (Anti-Mullerian Hormone (AMH); Cloud-Clone^®^ Corporation, Katy, TX, USA).

Each EIA was biochemically validated for *Oryctolagus cuniculus* and SP by verifying precision (intra-assay coefficients of variation (CV) from duplicated samples), sensitivity (smallest concentration analyzed), specificity (linearity of dilution), and accuracy (spike-and-recovery test).

### 2.6. Sperm Cryopreservation

The conventional freezing process was carried out in two steps. In the first step, 0.6 mL of phase A (when using Gent, without DMSO) of the dilution medium was added at room temperature and then the tubes were cooled to 5 °C for 120 min. In the second step, the same volume (0.6 mL) of phase B of the Gent extender or the corresponding freezing extender (TFC, GALAP), all with 10% DMSO, at 5 °C, were added and the samples were equilibrated at 5 °C for 2 h [61].

The samples were packed in 0.25-mL straws (*n* = 4 per freezing medium) containing 25 × 10^6^ spermatozoa/straw. The straws were sealed with polyvinyl alcohol (PVA) powder. Then, the straws were placed 5 cm above liquid nitrogen (LN_2_) and plunged into LN_2_ after 10 min (−196 °C). The samples were stored in LN_2_ until analysis. For thawing, the straws were immersed in a temperature-controlled bath at 37 °C for 30 s. The samples were analyzed after 10 min at 37 °C.

### 2.7. Post-Thawing Sperm Analyses

#### 2.7.1. Subjective Motility

A 10 μL drop of each sample was prepared on a slide warmed to 37 °C and covered with a coverslip. The assessment of subjective motility was performed in five fields at 200× magnification by using a phase-contrast microscope (M60i, Proiser, Spain) and the percentage of motile sperm was estimated over the total number of sperm observed.

#### 2.7.2. Concentration

Determination of sperm concentration was performed in a Neubauer-improved cell counting chamber or hemocytometer. After dilution of the samples (1:100) in distilled water, counting was carried out on a bright-field microscope at 400× magnification (BA310 Compound, Motic, Spain). To calculate the concentration, expressed as sperm/mL, the following formula was applied:

(sperm count × 1000)/(surface area × chamber depth × dilution) = sperm/mL

#### 2.7.3. Viability and Acrosomal Integrity

The samples were prepared as indicated below and, then, a 10 µL drop of each stained sample was added to a slide, covered with a coverslip, and examined under the fluorescence microscope (Nikon Eclipse TE2000-S Inverted Microscope, Nikon, Spain) except for the eosin-nigrosin stain.

a. Membrane integrity by eosin-nigrosin staining: a sample of thawed sperm was diluted to eosin-nigrosin stain on a slide (1:1), performed immediately after a thin smear. This vital stain distinguishes sperm with an altered plasma membrane in pink color, whereas live sperm do not stain. Sperm viability was evaluated in a bright field microscope (BA310 Compound, Motic, Spain) and at least five fields were counted at 400× magnification. The percentage of sperm with intact plasmalemma (eosin -) was calculated over the total number of sperm registered;

b. Plasma membrane integrity by propidium iodide (PI) staining (10^6^ sperm cells were stained with 2.4 µM PI (final concentration)): this technique that allows the identification of dead spermatozoa by staining the nucleus with red fluorescence (damaged plasma membrane). The percentage of sperm with intact plasma membrane (PI -) was calculated over the total number of sperm observed (minimum of 200 sperm cells per group);

c. Acrosome reacted by peanut agglutinin (PNA) lectin and PI staining (10^6^ sperm cells were stained with 2.4 µM PI and 1 µg/mL PNA (final concentrations)): a procedure for determining the status of the sperm acrosome is the PNA lectin labeling. The percentage of sperm with a damaged acrosome (PNA+) was calculated over the total number of sperm registered (minimum of 200 sperm cells per group);

d. Viability by SYBR-14 green and PI staining (10^6^ sperm cells were stained with 2.4 µM PI and 100 nM SYBR-14 (final concentrations)): SYBR-14 stains the nuclei of living sperm bright green. Conversely, PI stains only dead sperm (lack of membrane integrity). The percentage of sperm with an intact membrane (SYBR-14+, PI -) was calculated over the total number of sperm registered (minimum of 200 sperm cells per group).

#### 2.7.4. Mitochondrial Status

The samples were prepared as indicated below and, then, a 10 µL drop of each stained sample was added to a slide, covered with a coverslip, and examined under a fluorescence microscope (Nikon Eclipse TE2000-S Inverted Microscope, Nikon, Spain). A validation was performed for all the mitochondrial probes used in the study.

a. Mitochondrial potential by JC-1 staining (10^6^ sperm cells were stained with 1.5 nM JC-1 (final concentrations)): at low concentrations (due to low mitochondrial membrane potential), JC-1 is predominantly a monomer that yields green fluorescence with emission of 530 ± 15 nm. At high mitochondrial membrane potential, the dye aggregates yield a red–orange emission (590 ± 17.5 nm). The percentage of sperm with high membrane potential (JC-1 orange) was calculated over the total number of sperm registered (minimum of 200 sperm cells per group);

b. Mitochondrial activation by MitoTracker^TM^ Deep Red (MTDR) staining (10^6^ sperm cells were stained with 100 nM MTDR (final concentrations)): this fluorescent probe is a nontoxic, carbocyanine-based, far-red emission that is routinely used to chemically mark and visualize active mitochondria in living cells. Active mitochondria exhibit brighter fluorescence compared to inactive or damaged mitochondria. The percentage of sperm with high membrane potential (MTDR+) was calculated over the total number of sperm registered (minimum of 200 sperm cells per group).

#### 2.7.5. ProAKAP4 Analysis

According to the manufacturer’s instructions of the Rabbit 4MID^®^ Kit (SPQUI, S.A.S. Lille, France) used for the proAKAP4 analysis, approximately 50 × 10^6^ spermatozoa were resuspended in the kit lysis buffer and vortexed for 1 min at maximum speed. Then, after dilution with the buffer, the samples and the standard solutions (to perform the standard curve) were loaded into a 96-well plate precoated with proAKAP4 capture antibody and incubated for 2 h at room temperature on constant shaking (300 rpm). After extensive washing, the detection antibody was added and incubated for 1 h. After a second washing step, the detection substrate was added and incubated for 10 min. Then, the reaction was stopped and the optical density (450 nm) was immediately determined. A calculation data sheet provided by the manufacturer directly calculates the proAKAP4 as ng/mL per 10 × 10^6^ spermatozoa. A validation of the Rabbit 4MID^®^ Kit was performed specifically for rabbit sperm in the study and it is property of the commercial trademark.

### 2.8. Statistical Analyses

Data were analyzed to verify the normal distribution as well as the homogeneity of variances (homoscedasticity), using the Shapiro–Wilk and Levene’s statistical tests. The arcsine (x) transformation was used to normalize variables that did not meet conditions. Statistical analysis was carried out in R software version 3.6.1. with linear mixed-effect models (nlme package) and carrying out pairwise comparisons (multcomp package). The treatment groups (experimental groups) were used as fixed effects and the different individuals (*n* = 5) as the random component of the model for each individual experiment (Experiments 1 and 2). Additionally, correlations between proAKAP4 concentrations and variables analyzed in Experiments 1 and 2 were determined. Data are presented as mean ± standard error of the mean (SEM). Statistical significance was established at *p* < 0.05.

## 3. Results

### 3.1. Fresh Sperm-Quality Analyses

In Table 2 are shown the descriptive statistics for fresh sperm samples for Experi-ments 1 and 2, including total motility, membrane Integrity, viability, plasma mem-brane integrity, reacted acrosome mitochondrial membrane potential, and mitochon-drial activation,

### 3.2. SP Analyses

#### 3.2.1. Validation of the EIA: Cortisol, Testosterone, and AMH

Intra-assay CVs were 6.36 ± 1.12%, 3.36 ± 0.76%, and 4.96 ± 0.84%, for cortisol, testosterone, and AMH, respectively, all within the maximum accepted level. The sensitivities of the EIA kits were 40 pg/mL, 2 pg/mL, and 937.5 pg/mL for cortisol, testosterone, and AMH, respectively. The specificity test showed a correlation between the expected and observed levels with R^2^ of 0.99, 0.99, and 0.92 respectively, for cortisol, testosterone, and AMH (*p* < 0.05). The results of the spike-and-recovery test to measure accuracy presented a mean recovery percentage of 118.70%, 124.75%, and 118.01%, respectively for cortisol, testosterone, and AMH. The results obtained demonstrate that these EIA kits are strongly precise, specific, accurate, and sensible for quantifying cortisol, testosterone, and AMH concentrations.

#### 3.2.2. Cortisol, Testosterone, and AMH Level Analyses of SP

The following hormonal values were found in SP (Supplementary Figure S1): Experiment 1: 370.13 ± 224.58 pg/mL cortisol, 153.89 ± 35.77 pg/mL testosterone, and 6910.56 ± 975.82 pg/mL AMH in SP. For Experiment 2, the values were 161.75 ± 136.92 pg/mL cortisol, 111.02 ± 36.35 pg/mL testosterone, and 8447.97 ± 896.22 pg/mL AMH in SP.

### 3.3. Honey Addition of 2.5% Improves the Post-Thawing Motility but Not the Viability (Experiment 1)

**Figure 1:**
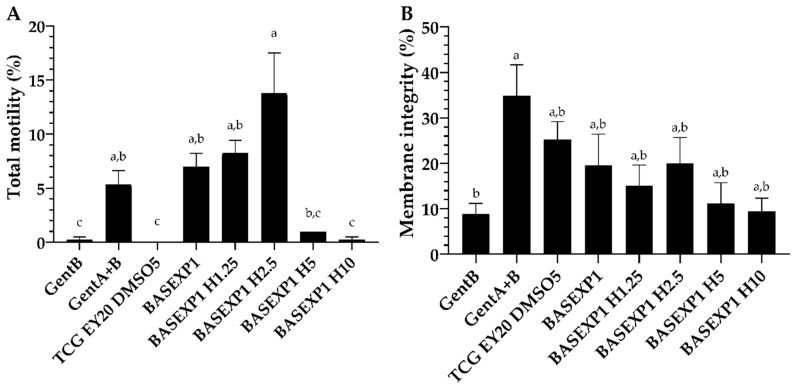
Rabbit-sperm quality parameters after cryopreservation using bee honey (H) as an egg-yolk replacement, in Experiment 1. Total motility ((**A**); %), and membrane integrity ((**B**); % eosin–spermatozoa). Gent B (Egg-yolk-based commercial extender); Gent A + Gent B (Egg-yolk-based commercial extender); TCG EY20 DMSO5 (Tris + citric acid + glucose + egg yolk (20%) + DMSO (10%); BASEXP1 (GALAP + DMSO (10%)); BASEXP1 H1.25 (GALAP + DMSO (10%) + H (1.25%); BASEXP1 H2.5 (GALAP + DMSO (10%) + H (2.5%)); BASEXP1 H5 (GALAP + DMSO (10%) + H (5%)); and BASEXP1 H10 (GALAP + DMSO (10%) + H (10%)). Mean ± SEM. Letters indicate significant differences among experimental groups for each studied parameter (*p <* 0.05).

The results indicate a significant reduction in both the motility and the viability of the sperm after the freezing–thawing process compared to fresh ejaculate. In addition, more than 2.5% of honey reduced significantly (*p* < 0.05) the motility of the samples (Figure 1).

**Figure 2:**
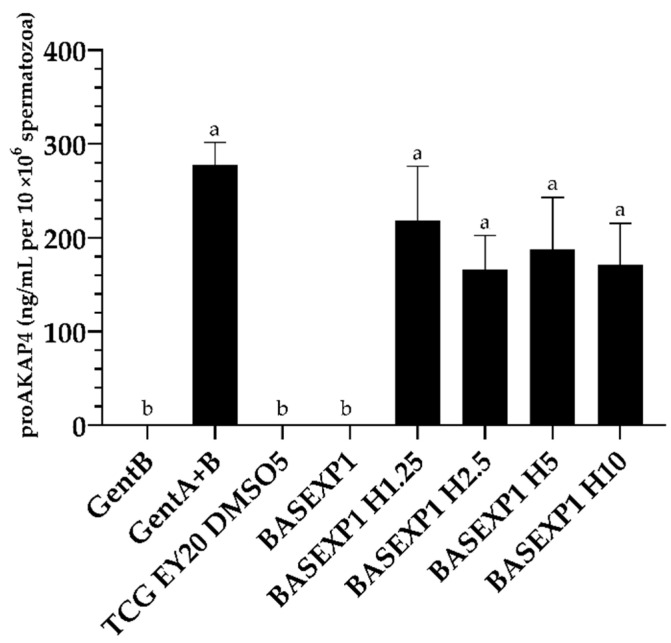
ProAKAP4 concentrations (ng/mL per 10 × 10^6^ spermatozoa) after rabbit spermcryopreservation using bee honey (H) as an egg-yolk replacement, in Experiment 1. Gent B (Egg-yolk-based commercial extender); Gent A + Gent B (Egg-yolk-based commercial extender); TCG EY20 DMSO5 (Tris + citric acid + glucose + egg yolk (20%) + DMSO (10%); BASEXP1 (GALAP + DMSO (10%)); BASEXP1 H1.25 (GALAP + DMSO (10%) + H (1.25%); BASEXP1 H2.5 (GALAP + DMSO (10%) + H (2.5%)); BASEXP1 H5 (GALAP + DMSO (10%) + H (5%)); and BASEXP1 H10 (GALAP + DMSO (10%) + H (10%)). Mean ± SEM. Letters indicate significant differences among experimental groups (*p <* 0.05).

Levels of proAKAP4 were conserved in the different honey concentrations and the commercial Gent A+B extender used in Experiment 1 (Figure 2, *p* < 0.05) but no levels of proAKAP4 were found in the base extender (GALAP + 5% DMSO), Gent B, and TCG + 20% egg yolk + 5% DMSO. There were no correlations in proAKAP4 concentrations and Experiment 1 analyzed variables.

### 3.4. CoQ10 Addition Is Deleterious for the Sperm Cryopreservation, Whereas No Effect Has Been Detected in the β-Carotene/α-Tocopherol Treatment Groups (Experiment 2)

**Figure 3:**
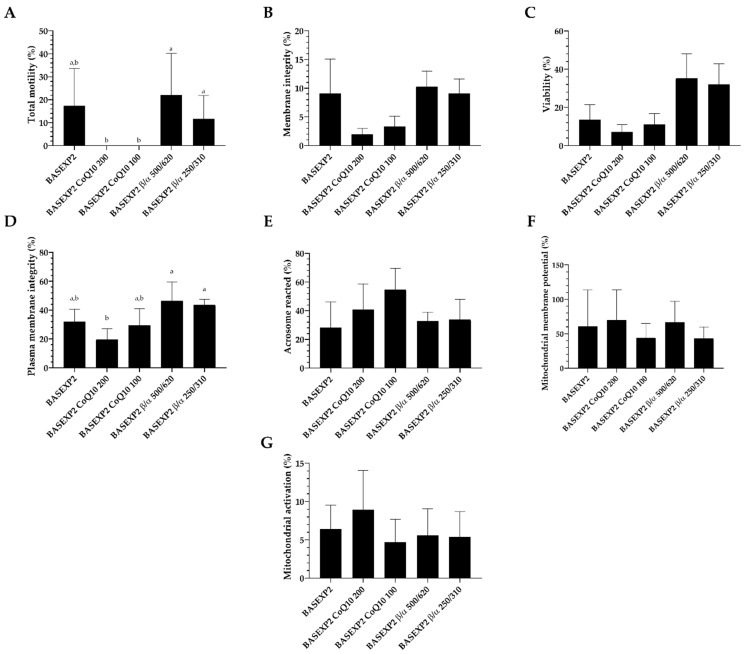
Rabbit sperm quality parameters after cryopreservation using antioxidants (CoQ10, and β-carotene/α-tocopherol), in Experiment 2. Total motility ((**A**); %), membrane integrity ((**B**); (%; eosin -), viability ((**C**); %; SYBR-14+), plasma membrane integrity ((**D**); %; PI -), acrosome reacted ((**E**); %; PNA+), mitochondrial membrane potential ((**F**); (%; JC-1 orange), mitochondrial activation (%; MTDR +; (**G**)). BASEXP2 (GALAP + egg-yolk (20%) + DMSO (10%)); BASEXP2 CoQ10 200 (GALAP + egg yolk (20%) + DMSO (10%) + CoQ10 (200 μM)); BASEXP2 CoQ10 100 (GALAP + egg-yolk (20%) + DMSO (10%) + CoQ10 (100 μM)); BASEXP2 β/α 500/620 (GALAP + egg yolk (20%) + DMSO (10%) + β/α (500 μM/620 μM)); and BASEXP2 β/α 250/310 (GALAP + egg-yolk (20%) + DMSO (10%) + β/α (250 μM/310 μM)). Mean ± SEM. Letters indicate significant differences among experimental groups for each studied parameter (*p <* 0.05). Absence of letters means nonstatistical differences (*p >* 0.05).

The results from Experiment 2 showed a complete reduction of sperm movement, independently of the concentration of CoQ10 used (Figure 3A). Moreover, both CoQ10 groups failed to improve the membrane integrity post-thawing (Figure 3B). No differences were found in viability or the percentage of acrosomes reacted (Figure 3C,E). However, the higher concentration of CoQ10 significantly (*p* < 0.05) reduced the plasma membrane integrity, relative to the β-carotene/α-tocopherol treatments (Figure 3D). No differences were found among groups in the mitochondrial membrane potential and in the mitochondrial activity analyses (Figure 3F,G).

**Figure 4:**
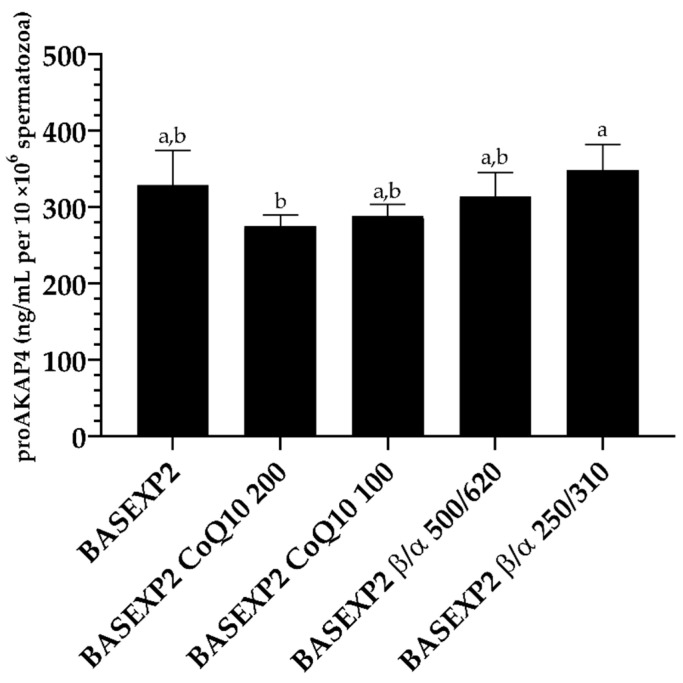
ProAKAP4 concentrations (ng/mL per 10 × 10^6^ spermatozoa) after rabbit sperm thawing in Experiment 2. BASEXP2 (GALAP + egg yolk (20%) + DMSO (10%)); BASEXP2 CoQ10 200 (GALAP + egg-yolk (20%) + DMSO (10%) + CoQ10 (200 μM)); BASEXP2 CoQ10 100 (GALAP + egg-yolk (20%) + DMSO (10%) + CoQ10 (100 μM)); BASEXP2 β/α 500/620 (GALAP + egg-yolk (20%) + DMSO (10%) + β/α (500 μM/620 μM)); and BASEXP2 β/α 250/310 (GALAP + egg-yolk (20%) + DMSO (10%) + β/α (250 μM/310 μM)). Mean ± SEM. Letters indicate significant differences among experimental groups (*p* < 0.05).

The levels of proAKAP4 in Experiment 2 were decreased in the 200 μM CoQ10 supplementation group compared to the 250 μM/310 μM β-carotene/α-tocopherol supplementation group (Figure 4, *p* < 0.05). Additionally, there were correlations of proAPAK4 with mitochondrial membrane potential (R^2^ = 0.29, *p* = 0.04) and mitochondrial activation (R^2^ = 0.35, *p* = 0.02) (Supplementary Figure S2).

## 4. Discussion

The concentration of sperm obtained in the present study (360.54 ± 54.57 × 10^6^ spermatozoa/mL) falls within the range of sperm concentration in the rabbit, usually between 50 and 500 × 10^6^ sperm/mL [1]. In most studies, the ejaculates that are considered suitable after performing a macroscopic and microscopic evaluation are mixed to obtain a heterospermic pool that compensates for the ejaculates with a lower concentration [62]. In our study, all the samples were centrifuged to separate the SP. Despite having specific protective effects on the sperm membranes [63], SP can affect the motility of these cells, so their elimination is ultimately beneficial [64]. The motility and viability standards described in rabbits must be above 80% and 70%, respectively [65]. In our results, all the fresh samples met these requirements, considering that these standards have been defined for mixed ejaculate samples from various animals and not for using individually processed ejaculates. The present study included a total of five adult rabbit bucks in which the sperm-quality parameters were evaluated both fresh and after using three different types of additives to the cryopreservation extender media: honey, CoQ10, and β-carotene/α-tocopherol, by using a conventional freezing procedure.

Sperm cryopreservation induces changes in membrane integrity, decreasing sperm quality and, thus, impairing their fertilizing ability [61]. Sperm is particularly sensitive to oxidative stress due to the high content of unsaturated fatty acids [66], causing reduced sperm motility [67], loss of viability, and acrosomal integrity [68], among other deleterious effects. In our study, the cryopreservation process reduced the motility compared to the values of fresh semen, in agreement with previous results from Moce and Vicente [3], highlighting the detrimental effects of cryopreservation on motile and live sperm populations. Interestingly, the supplementation with 2.5% honey (about 110 mM glucose/fructose [69]) showed higher motility than the higher concentrations and with a promising effect with respect to the control, which could be clarified in a follow-up study. Honey is a complex substance [13] and its composition and biological activities vary according to the geographical and floral source used by honeybees [70]. Previous studies have used honey as a semen extender component in several species [15,16,17,18,19,20,21,22], and some as an alternative to antibiotics for cryopreservation. Similar to our results, concentrations of greater than 2.5% honey decreased the post-thaw motility in rams [17] and bulls [20,21]. Our findings, however, differ from those of Fakhrildin and collaborators [18], who reported better postsperm quality at 10% honey supplementation in human sperm, which may have higher resilience to high osmotic environments. We have shown that honey supplementation increases the osmolality (nonpermeable component), which may have produced an excessively hyperosmotic extracellular environment, causing excessive spermatozoa dehydration and, ultimately, cell death [21]. In this regard, we can attribute the loss of motility in the egg-yolk commercial extender Gent B and the two extenders containing CoQ10 to the higher osmolality observed in these groups.

Sperm (spermatozoa and SP) have a limited antioxidant capacity [61]. Thus, antioxidant supplementation of sperm extenders for cryopreservation purposes has been extensively used in diverse animal species [71] but, to the best of our knowledge, only one study has been applied to the rabbit species [61]. This research study, performed by Maya-Soriano et al., 2013 [61], used bovine-serum albumin, retinol, and retinyl, all of them failing to improve the sperm quality after cryopreservation. In other species, when antioxidants are added to the semen extender, the sperm’s ability to survive freezing and thawing is improved [11]. Indeed, CoQ10 has been proposed as a supplement to freezing extenders to improve the sperm quality of stallions [34,35,36,37], buffalo and cattle [72], rams [73], bucks [74], and roosters [75]. However, CoQ10 may not be adequate for freezing rabbit spermatozoa, at least at the range tested in this study, which are detrimental to the motility of rabbit spermatozoa and seemed to decrease the plasma membrane integrity in the higher concentration used. One plausible explanation for this is the use of ethanol for preparing the CoQ10 solution. The maximum solubility ratio of the CoQ10 is adjusted to 1 mg/mL of ethanol, being the final concentration of ethanol of 8.6% and 4.3%, in group CoQ10 200 µM and CoQ10 100 µM, respectively. Further research is needed to evaluate if lower CoQ10 concentrations may exert a positive effect on rabbit-sperm cryopreservation, thus reducing the high osmotic pressure measured in the media containing CoQ10.

Regarding the last two antioxidants used in our experimental setup, β-carotene and α-tocopherol have been used as supplements in rainbow-trout sperm extenders [57] and α-tocopherol for cryopreservation of bull semen [58]. However, our results demonstrated that β-carotene/α-tocopherol addition to the cryopreservation extender did not affect post-thaw motility in rabbit sperm but seemed to improve viability.

Finally, our study included the analysis of proAKAP4 concentration since it has been described as a functional marker of post-thawed spermatozoa in several species (equine [53,54]; bovine [55]; and ovine [56]). This study is, to the best of our knowledge, the first to use proAKAP4 assessment in rabbit spermatozoa frozen with different extender compositions. However, more studies should be performed in order to test the applicability of the proAKAP4 as a cryopreservation biomarker in rabbits, requiring further validation by fertility trials to test if the results collected here will reflect a differential fertility performance.

## 5. Conclusions

The cryopreservation protocols, including the use of egg-yolk replacements, such as honey, and the addition of antioxidants (CoQ10, β-carotene, and α-tocopherol) used in this study, failed to improve the sperm-quality parameters of rabbit spermatozoa after cryopreservation. The development of new strategies oriented towards the improvement of species-specific cryopreservation protocols is nowadays a great challenge when it comes to rabbits. In particular, any improvement would allow sperm to increase their survival after the cryopreservation process, with potential application both at an experimental level and in animal production. In this sense, this study suggested that proAKAP4 could be a potential marker for sperm cryopreservation in rabbits.

## Figures and Tables

**Table 1:** Extender composition and osmolality used in Experiments 1 and 2.

	Extender	Composition	Osmolality (mOsm/kg)
Experiment 1	Gent B	Egg-yolk-based commercial extender	Higher ^$^
	Gent A + Gent B	Egg-yolk-based commercial extender	749
	TCG EY20 DMSO5	Tris + citric acid + glucose + EY (20%) + DMSO (10%) *	1110
	BASEXP1	GALAP + DMSO (10%) *	1133
	BASEXP1 H1.25	GALAP + DMSO (10%) + H (1.25%) *	1172
	BASEXP1 H2.5	GALAP + DMSO (10%) + H (2.5%) *	1183
	BASEXP1 H5	GALAP + DMSO (10%) + H (5%) *	1407
	BASEXP1 H10	GALAP + DMSO (10%) + H (10%) *	1850
Experiment 2	BASEXP2	GALAP + EY (20%) + DMSO (10%) *	1171
	BASEXP2 CoQ10 200	GALAP + EY (20%) + DMSO (10%) + CoQ10 (200 μM) *	Higher ^$^
	BASEXP2 CoQ10 100	GALAP + EY (20%) + DMSO (10%) + CoQ10 (100 μM) *	Higher ^$^
	BASEXP2 β/α 500/620	GALAP + EY (20%) + DMSO (10%) + β/α (500 μM/620 μM) *	1184
	BASEXP2 β/α 250/310	GALAP + EY (20%) + DMSO (10%) + β/α (250 μM/310 μM) *	1160

Gent^®^ (Minitube Ibérica, Spain), GALAP (IMV technologies, L’Aigle, France), EY (egg yolk), DMSO (dimethyl sulfoxide), CoQ10 (coenzyme Q10), β-α (β-carotene/α-tocopherol mixture), H (honey). All sperm samples were diluted 1:1 with their respective semen extender. * A first diluent (A) is made without the DMSO and a second one (B) with DMSO 10%, to reach a final concentration of 5% DMSO. ^$^ The measurement is higher than the upper limit detection of the osmometer.

**Table 2:** Fresh sperm-quality parameters analyzed in rabbit-buck ejaculates (*n* = 5). Data are represented as mean ± SEM for samples in Experiments 1 and 2.

Experiment	Total Motility (%)	Membrane Integrity (%)	Viability (%)	Plasma Membrane Integrity (%)	Acrosome Reacted (%)	Mitochondrial Membrane Potential (%)	Mitochondrial Activation (%)
1	60.0 ± 9.7	68.2 ± 10.2	-	-	-	-	-
2	53.3 ± 11.6	57.5 ± 13.5	72.1 ± 13.5	62.9 ± 12.2	19.9 ± 6.6	38.6 ± 13.3	10.9 ± 9.0

Total motility (%), membrane integrity (%; eosin -), viability (%; SYBR-14+), plasma membrane integrity (%; PI -), acrosome reacted (%; PNA+), mitochondrial membrane potential (%; JC-1 orange), and mitochondrial activation (%; MTDR+). Mean ± SEM.

## Data Availability

The data presented in this study are available in this article.

## Supplementary Materials

The following are available online at https://www.mdpi.com/article/10.3390/ani13142392/s1, Supplementary Figure S1. Hormone-level analyses in seminal plasma from animals included in Experiments 1 and 2.; Supplementary Figure S2. Correlations of proAKAP4 levels and mitochondrial membrane potential and activation from animals included in Experiment 2.
