# Supplementary material for: Effect of Honey, Coenzyme Q10, and β-Carotene/α-Tocopherol as Novel Additives in Rabbit-Sperm Cryopreservation Extender
Source: Animals (Basel). 2023 Jul 24;13(14):2392. doi: 10.3390/ani13142392 (PMC10376550; doi:10.3390/ani13142392)
Supplement: Supplementary file 1 [file animals-13-02392-s001.zip › animals-2433261-supplementary.pdf]

Supplementary files

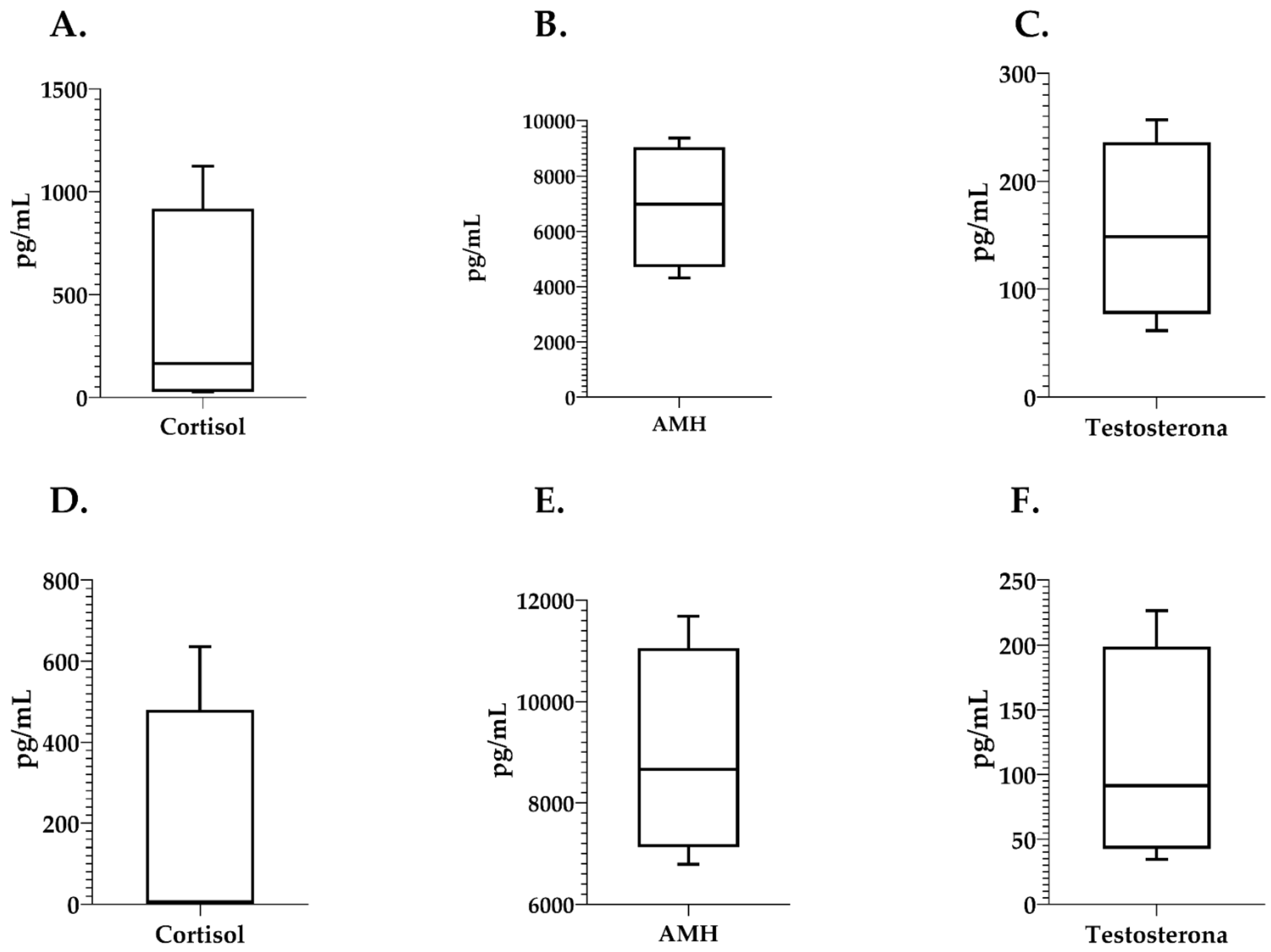

Scheme 1. Hormone levels analyses in seminal plasma from animals included in Experiment 1 (A-C) and Experiment 2 (D-F).

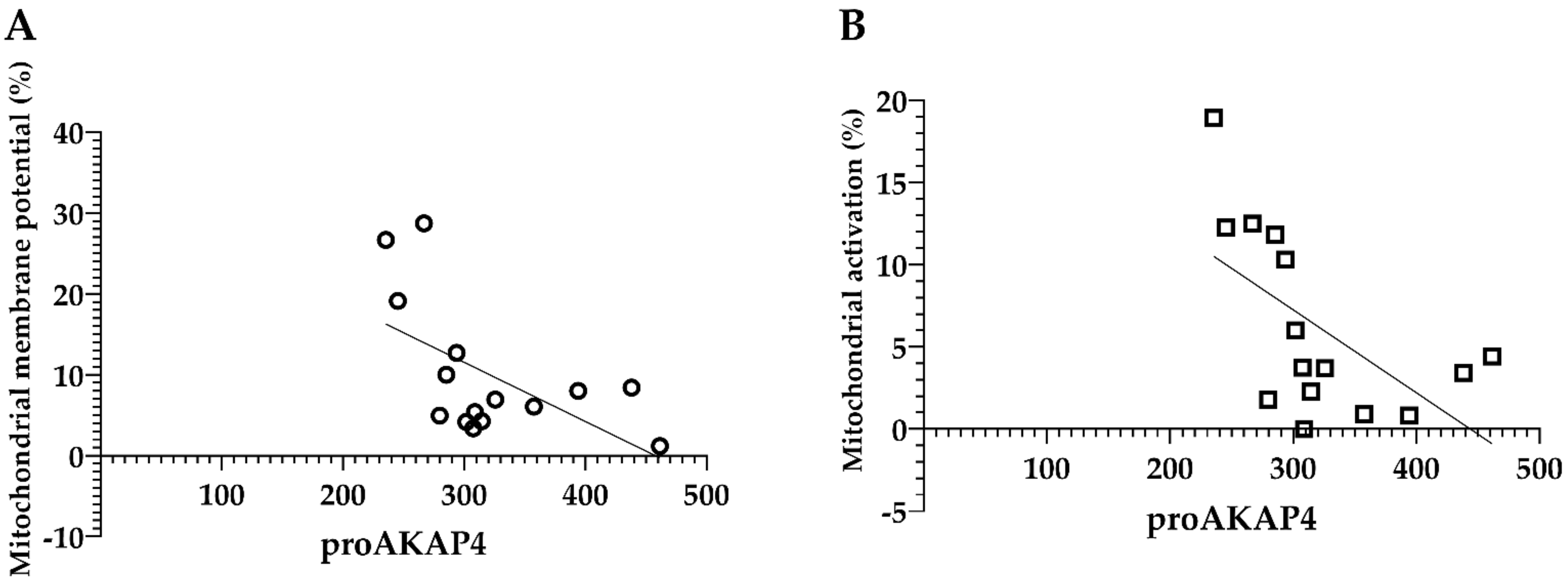

Supplementary Figure S2. Correlations of proAKAP4 levels and mitochondrial membrane potential and activation.
